# Sex Specific Gene Regulation and Expression QTLs in Mouse Macrophages from a Strain Intercross

**DOI:** 10.1371/journal.pone.0001435

**Published:** 2008-01-16

**Authors:** Jeffrey M. Bhasin, Enakshi Chakrabarti, Dao-Quan Peng, Aneesh Kulkarni, Xi Chen, Jonathan D. Smith

**Affiliations:** 1 Department of Cell Biology, Cleveland Clinic, Cleveland, Ohio, United States of America; 2 Department of Quantitative Health Sciences, Cleveland Clinic, Cleveland, Ohio, United States of America; 3 Department of Molecular Medicine, Cleveland Clinic Lerner College of Medicine of Case Western Reserve University, Cleveland, Ohio, United States of America; University of Chicago, United States of America

## Abstract

**Background:**

A powerful way to identify genes for complex traits it to combine genetic and genomic methods. Many trait quantitative trait loci (QTLs) for complex traits are sex specific, but the reason for this is not well understood.

**Methodology/Principal Findings:**

RNA was prepared from bone marrow derived macrophages of 93 female and 114 male F_2_ mice derived from a strain intercross between apoE-deficient mice on the AKR and DBA/2 genetic backgrounds, and was subjected to transcriptome profiling using microarrays. A high density genome scan was performed using a mouse SNP chip, and expression QTLs (eQTLs) were located for expressed transcripts. Using suggestive and significant LOD score cutoffs of 3.0 and 4.3, respectively, thousands of eQTLs in the female and male cohorts were identified. At the suggestive LOD threshold the majority of the eQTLs were trans eQTLs, mapping unlinked to the position of the gene. Cis eQTLs, which mapped to the location of the gene, had much higher LOD scores than trans eQTLs, indicating their more direct effect on gene expression. The majority of cis eQTLs were common to both males and females, but only ∼1% of the trans eQTLs were shared by both sexes. At the significant LOD threshold, the majority of eQTLs were cis eQTLs, which were mostly sex-shared, while the trans eQTLs were overwhelmingly sex-specific. Pooling the male and female data, 31% of expressed transcripts were expressed at different levels in males vs. females after correction for multiple testing.

**Conclusions/Significance:**

These studies demonstrate a large sex effect on gene expression and trans regulation, under conditions where male and female derived cells were cultured ex vivo and thus without the influence of endogenous sex steroids. These data suggest that eQTL data from male and female mice should be analyzed separately, as many effects, such as trans regulation are sex specific.

## Introduction

The combination of quantitative trait locus (QTL) mapping and gene expression profiling allows for the identification of expression quantitative trait loci (eQTLs), which are loci associated with the expression of each transcript. This method was first applied to a yeast strain intercross, where both cis-acting and trans-acting loci were identified associated with the expression level of hundreds of transcripts [1]. eQTL analysis was applied to mouse tissues from an F_2_ cohort derived from a strain intercross yielding thousands of eQTLs, which were distributed non-randomly over the genome yielding hotspots that each contained hundreds of eQTLs [2]. eQTLs have also been described using human lymphoblastoid cell lines from defined pedigrees [2]–[4]. This methodology has been used, in so-called ‘genetical-genomics’ studies [5], as an aid to identify candidate genes for complex phenotypic traits, such as obesity, in mouse strain intercross studies [6]–[9]; and, it has been a major shortcut in the identification of QTL causative genes, for example the identification of ABCC6 as the gene responsible for dystrophic cardiac calcification in DBA/2 mice [10].

Sex specific effects are quite common in mouse studies, for example PPARγ agonist treatment reduces atherosclerosis lesion areas in male, but not female, LDL receptor-deficient mice [11]. Similarly, gene expression studies in male and female F_2_ mice have shown a large degree of sexually dimorphic gene expression in liver, adipose tissue, muscle, and to a lesser extent in brain [12], [13]. Mouse phenotypic QTLs, such as gonadal fat pad mass [12] or atherosclerotic lesion areas [14], [15], are also commonly sexually dimorphic, with many specific QTLs found in only male or female cohorts. Likewise, many mouse tissue eQTLs are also sexually dimorphic [12], [13]. Prior mouse eQTL studies employed freshly isolated tissues, thus, many sexually dimorphic effects on gene expression could be due to exposure to the different hormonal milieu in male and female mice. In the current study, we employed bone marrow derived macrophages from a mouse strain intercross that was cultured 2 weeks *ex vivo*. We still found that many eQTLs are sex specific, and remarkably, that 30% of expressed genes were differentially expressed in female vs. male macrophages, suggesting that a large extent of sexually dimorphic gene expression may be directly dependent on X and Y chromosome dosage, rather than on the hormonal environment.

## Results

### Suggestive eQTLs

Microarray (Affymetrix 430v2) gene expression data were obtained from bone-marrow derived macrophages of 93 female and 114 male F_2_ mice derived from a strain intercross between apoE-deficient mice on the AKR and DBA/2 backgrounds. Since gene expression in somatic mouse tissues is highly sex specific [12], [13], we analyzed eQTLs separately in males and females. We limited our analysis to transcripts that were expressed in at least 1/3 of the samples within each sex, using this liberal cut off so as not to omit transcripts that expressed in only one of the parental strains. The female sample had 21,798 expressed transcripts, with 17,986 (82%) of these transcripts expressed in at least 75% of the female samples. Due to presence of multiple probes for some genes, these 21,798 transcripts represent only 11,531 unique genes. We used the gene expression of each of these transcripts as a phenotype, along with a high density genome scan composed of 1,967 informative SNPs on a mouse SNP chip [15], to identify eQTLs associated with the expression level of each transcript in a genome-wide method of generating LOD plots for expression of each transcript across the mouse genome. We used an initial suggestive LOD cutoff of ≥3.0. With 2 degrees of freedom, this suggestive LOD threshold corresponds to a nominal p-value of 1×10^−3^ [16]. We calculated a genome wide p-value of 0.25 for this LOD threshold by 1000 permutations each of 10 randomly selected female eQTLs with a LOD score of 3.00. We identified a total of 9,308 eQTLs in the female mice that met this suggestive LOD threshold, and applying our genome wide p-value at this threshold, we expect ∼7000 of these eQTLs to be authentic. We characterized as cis eQTLs those in which the eQTL mapped on the same chromosome and within 20 Mb of the transcript location on the mouse genome. All of the remaining eQTLs were identified as either trans eQTLs (eQTL maps at a different locus than the transcript), or ambiguous eQTLs for which the Affymetrix probe target sequence matched to more than one genomic location. There were 1,859 cis eQTLs in the female mice, representing 20% of the total eQTLs, and their average LOD score was 8.77. There were 6,117 trans eQTLs in the female mice, representing 66% of the total eQTLs, and their average LOD score was 3.55. We also identified 1332 ambiguous eQTLs, representing 14% of the total eQTLs, with an average LOD score was 5.08. Table 1 provides a summary of the eQTL findings, and Supplemental Table S1 gives the details of each of the 9,308 female eQTLs, arranged by the genomic location of the eQTL.

**Table 1:** eQTL summary in female and male F_2_ mice

	Females		Males (autosomes, X Chr)		Males Y Chr	
	***Count***	***Ave. LOD***	***Count***	***Ave. LOD***	***Count***	***Ave. LOD***
**LOD 3.0**						
Total eQTLs	9,308	4.81	12,361	4.78	1,145	4.06
Cis eQTLs	1,859 (20.0%)	8.77	1,990 (16.1%)	9.46	4 (0.35%)	5.71
Trans eQTLs	6,117 (65.7%)	3.55	8,625 (69.8%)	3.64	1,026 (89.6%)	4.07
Ambiguous eQTLs	1,332 (14.3%)	5.08	1,746 (14.1%)	5.07	115 (10.0%)	3.96
**LOD 4.3**						
Total eQTLs	2,177	9.38	2,988	8.95	334	5.30
Cis eQTLs	1,321 (60.7%)	10.89	1,445 (48.4%)	11.67	2 (0.60%)	7.86
Trans eQTLs	526 (24.2%)	5.14	1,086 (36.4%)	5.06	309 (92.5%)	5.29
Ambiguous eQTLs	330 (15.1%)	10.13	457 (15.3%)	9.6	23 (6.9%)	5.34

A similar eQTL analysis was performed for the male cohort with 21,733 expressed transcripts (representing 11,557 unique genes), with 17,632 (81% of these transcripts) expressed in at least 75% of the male samples. For the 19 autosomes and the X chromosome, we identified 12,361 eQTLs with a LOD score of ≥3.0. We calculated a genome wide p-value of 0.25 for this LOD threshold by 1000 permutations each of 10 male eQTLs with a LOD score of 3.00. There were 1,990 cis eQTLs, representing 16% of the total male eQTLs, and their average LOD score was 9.46. There were 8,625 trans eQTLs, representing 70% of the total eQTLs, and their average LOD score was 3.64. There were also 1,746 ambiguous eQTLs, representing 14% of the total, with an average LOD score of 5.07. As our strain intercross used males from both strains, we also looked for eQTLs due to the grandparental Y chromosome, and we detected 1145 that met the LOD>3.0 threshold. 90% of these were trans eQTLs, 10% were ambiguous eQTLs, with only 4 potentially cis eQTLs, associated with probes mapping to the Y chromosome (Table 1). Supplemental Table S2 provides the details of each of the male eQTLs.

Overall, as seen in prior eQTL studies using a liberal LOD threshold [2], [12], there were many more trans eQTLs observed in both the female and male cohorts, but these had lower LOD scores than the cis eQTLs, presumably due to the more direct effect of cis variation in regulatory or transcribed regions on gene expression or mRNA stability.

### eQTL Hotspots

**Figure 1:**
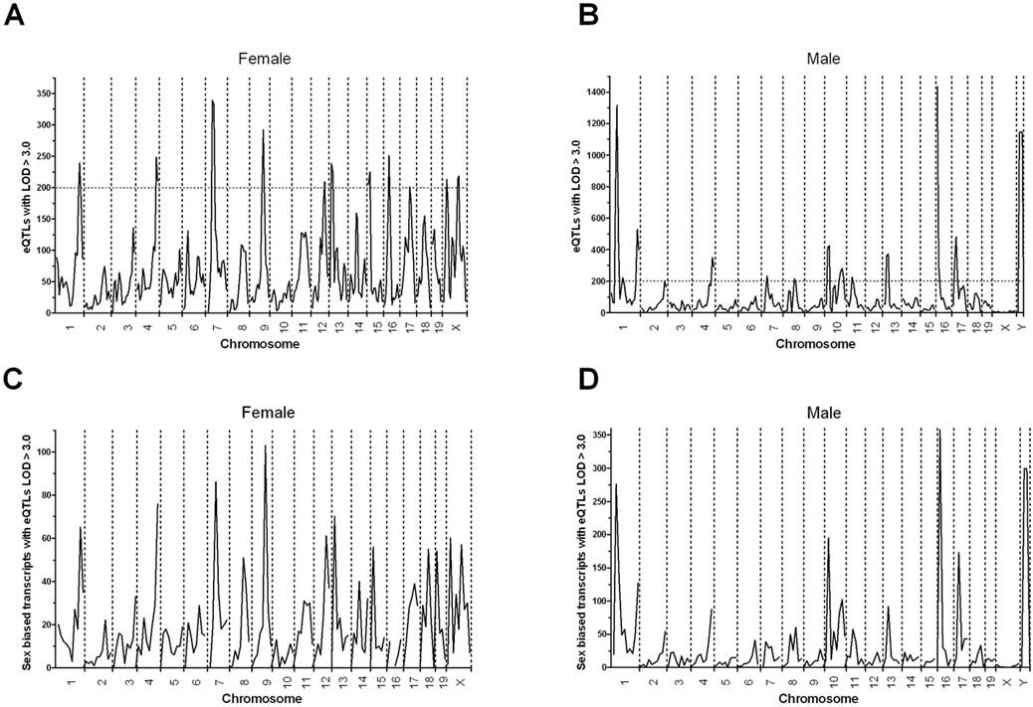
Genomic location of eQTLs. Bone marrow derived macrophage eQTLs with LOD≥3.0 were mapped to partially overlapping 20 Mb bins in female (A) and male (B) F_2_ mice. The horizontal dotted line denotes the arbitrary cutoff for eQTL hotspots. eQTLs for sex biased transcripts with LOD≥3.0 were mapped to partially overlapping 20 Mb bins in female (C) and male (D) F_2_ mice.

We examined the distribution of these suggestive eQTLs over the mouse genome in partially overlapping 20 Mb bins. There were non-random distributions in both female and male cohorts. In the female samples there were 11 hotspots of eQTLs, each having over 200 eQTLs (>2% of all female eQTLs), with the largest peak on chromosome 7 in a bin that had 339 eQTLs (Figure 1A). There were 2 hotspots on the X chromosome in the females, and overall there were 861 eQTLs on the X chromosome (11 cis, 698 trans, and 170 ambiguous). In the males there were 13 hotspots with over 200 eQTLs (Figure 1B). The male data yielded three super hotspots each containing between 8.5 to 11.5% of all the male eQTLs, one near the proximal end of chromosome 1 containing 1316 eQTLs, one on chromosome 16 containing 1434 eQTLs, and one for the entire Y chromosome containing 1145 eQTLs (Fig 1B). Gene ontology analysis did not detect over representation in any functional group of the transcripts in the chromosome 1 and 16 eQTL hotspots compared to all male eQTLs. For the transcripts associated with the Y chromosome eQTL hotspot compared to all eQTL associated transcripts on the autosomes and the X chromosome, the gene ontology analysis found several over represented classes including the chromosome and extracellular cellular components, the cell cycle M phase and DNA metabolic biological processes, and the microtubule motor activity molecular function (see Supplemental Table S3 for full list and P values). There was only limited overlap of the eQTL hotspot positions in females and males, near the distal ends of chromosome 1 and 4, and near the proximal end of chromosomes 7 and 13. Interestingly, there were only 46 eQTLs in the male cohort that mapped to the X chromosome (20 cis, 11 trans, and 15 ambiguous), showing an unexpectedly large difference compared to the female cohort where 861 eQTLs mapped to the X chromosome.

In order to identify potential candidates responsible for the two male autosomal super hotspots, we examined cis eQTLs that mapped to these hotspots and had a LOD score of ≥5.5. We then correlated the expression levels of each of these cis eQTLs with the expression of the trans eQTLs that mapped precisely to the most common marker for trans eQTLs in these region. We found nine cis eQTLs probes on chromosome 1 whose expression had an average absolute value correlation coefficient >0.20 with 308 trans eQTLs at that locus; and, we found three chromosome 16 cis eQTLs whose expression had an average absolute value correlation coefficients >0.2 with 379 trans eQTLs at that locus. Each of these cis eQTLs, listed in Tables 2 and 3, respectively, are candidate genes whose expression is strain dependent that could be responsible for mediating the trans regulation of ∼1000 other genes.

**Table 2:** Correlations of expression data for chromosome 1 hotspot cis eQTLs with 308 trans eQTLs in males.

*Probe*	*Probe mega-base*	*Marker mega-base*	*LOD*	*Average Correlation (R)*	*Gene Name*	*Description*
1435475_at	36.37	46.54	10.71	0.30	Lman2l	lectin, mannose-binding 2-like
1417293_at	36.01	48.59	6.36	0.29	Hs6st1	heparan sulfate 6-O-sulfotransferase 1
1416931_at	58.39	52.97	8.43	0.26	Nif3l1	Ngg1 interacting factor 3-like 1 (S. pombe)
1459679_s_at	51.69	51.87	7.60	−0.24	Myo1b	myosin IB
1436984_at	60.42	58.35	14.01	−0.24	Abi2	abl-interactor 2
1434422_at	57.33	58.35	8.18	0.23	Unknown	Unknown
1434303_at	60.43	48.59	9.90	−0.21	Raph1	Ras association (RalGDS/AF-6) and pleckstrin homology domains 1
1428425_at	42.99	48.59	7.60	0.21	Tgfbrap1	transforming growth factor, beta receptor associated protein 1
1421982_a_at	37.37	55.71	6.60	−0.21	Unc50	unc-50 homolog (C. elegans)

(Only probes with average expression value greater than 150 used in correlations)

**Table 3:** Correlations of expression data for chromosome 16 hotspot cis eQTLs with 379 trans eQTLs in males.

*Probe*	*Probe mega-base*	*Marker mega- base*	*LOD*	*Average Correlation (R)*	*Gene Name*	*Description*
1435969_at	3.89	7.64	6.09	0.34	Btbd12	BTB (POZ) domain containing 12
1435439_at	18.17	7.64	5.55	0.31	Dgcr8	DiGeorge syndrome critical region gene 8
1437524_x_at	4.54	5.27	8.92	−0.22	Coro7	coronin 7

### Suggestive eQTL Sharing Between the Sexes

We examined the suggestive eQTLs on the autosomes and the X chromosome to determine how many were shared between the female and male cohorts, thus the identical probe had an eQTL in both sexes that mapped within 20 Mb to the same locus (Table 4). 1,285 cis eQTLs were shared, representing 69% of the female and 65% of the male cis eQTLs (Supplemental Table S4). Among these sex-shared cis eQTLs, the LOD scores for the female eQTLs were highly correlated with the LOD scores in males (r^2^ = 0.71, p<0.0001), highlighting the similarity of these shared eQTLs. A very different picture emerged from the trans eQTLs, with only 71 being shared between the sexes, representing only 1.2% and 0.8% of the female and male trans eQTLs, respectively. Supplemental Table S5 details each of these 71 shared trans eQTLs that had mean LOD scores of 4.07 and 4.13 in the female and male cohorts, respectively. Among these sex-shared trans eQTLs, a correlation between female and male LOD scores was evident (r^2^ = 0.26, p<0.0001), but was weaker than that observed for the sex shared cis eQTLs (r^2^ = 0.71). There was some clustering of these sex-shared trans eQTLs, with 7 mapping to the proximal end of chromosome 1, 11 mapping to the distal end of chromosome 1, and 7 mapping to the distal end of chromosome 4. The gene ontology associations of the sex-shared trans eQTLs were statistically compared against all of the trans eQTLs. This analysis yielded no apparent clustering based on known gene functions, or any particular pathway in the sex shared trans eQTLs. There were also 243 sex-shared ambiguous eQTLs with very high average LOD scores (11.35 and 12.7 in females and males, respectively), indicating that most of these ambiguous eQTLs were actually cis eQTLs, but their probes were not uniquely assigned to the mouse genome (Supplemental Table S6).

**Table 4:** eQTLs shared by male and female F_2_ mice.

	***Count***	***Ave. LOD female***	***Ave. LOD male***	***% female shared***	***% male shared***
**LOD 3.0**					
Total shared eQTLs	1,599	10.31	13.17	17.2	13.0
Cis shared eQTLs	1,285 (80.2%)	10.47	11.90	69.1	64.6
Trans shared eQTLs	71 (4.6%)	4.07	4.13	1.2	0.82
Ambiguous shared eQTLs	243 (15.2%)	11.35	12.70	18.2	13.9
**LOD 4.3**					
Total shared eQTLs	1,151	12.65	14.36	52.9	38.5
Cis shared eQTLs	965 (83.8%)	12.45	14.09	73.1	66.8
Trans shared eQTLs	9 (0.8%)	5.97	6.82	1.7	0.83
Ambiguous shared eQTLs	177 (15.4%)	14.05	16.21	53.6	38.6

### Significant eQTLs

We repeated the eQTL analysis using a significant LOD threshold of 4.3, yielding a nominal p value of 5×10^−5^ [16]. Permutation analysis revealed that the LOD 4.3 threshold yielded a genome wide p-value of 0.02 in both males and females. In the females, 2,177 eQTLs met this LOD threshold, with 1,321 cis eQTLs, representing 61% of the total eQTLs, and 526 trans eQTLs, representing 24% of the total eQTLs (Table 1). There were also 330 ambiguous eQTLs (15% of the total eQTLs) that had a very high average LOD score of 10.13. Again, this indicates that the majority of these ambiguous eQTLs were actually due to strongly associated cis eQTLs, but their probes were not uniquely assigned to the mouse genome. Of the suggestive female cis eQTLs that met the LOD>3.0 threshold, 71% met the LOD 4.3 threshold cutoff, while only 8.6% of the female suggestive trans eQTLs were maintained at this LOD stringency, again indicating the relative strength of the cis eQTLs compared to the trans eQTLs.

In the males, 2,988 eQTLs met the LOD 4.3 threshold that mapped to the autosomes and the X chromosome, with 1,445 cis eQTLs, representing 48% of the total eQTLs, and 1,086 trans eQTLs, representing 36% of the total eQTLs (Table 1). There were also 457 ambiguous eQTLs (15% of the total eQTLs) that had a very high average LOD score of 9.6. Of the suggestive male cis eQTLs that met the LOD>3.0 threshold, 73% met the LOD 4.3 threshold cutoff, while only 13% of the trans eQTLs were maintained at this LOD stringency. There were also 334 male eQTLs that mapped to the Y chromosome that met the LOD 4.3 threshold, with 30% of the LOD 3.0 threshold trans eQTLs maintained at the LOD 4.3 stringency, a higher retention rate than observed for the autosomal and X chromosome trans eQTLs. Thus in both male and female mice, this more stringent LOD threshold eliminated most of the trans eQTLs but retained most of the cis eQTLs.

### Significant eQTL Sharing Between the Sexes

We examined the LOD 4.3 threshold eQTLs on the autosomes and the X chromosome to determine how many were shared between the female and male cohorts (Table 4). 965 cis eQTLs were shared, representing 73% of the female and 67% of the male cis eQTLs at this stringency. Only nine trans eQTLs at this LOD threshold in both sexes were shared in females and males, representing 1.7% and 0.8% of the female and male trans eQTLs, respectively. These nine shared trans eQTLs had mean LOD scores of 5.97 and 6.82 in the female and male cohorts, respectively.

This very low level of sharing could be due to most of the trans eQTLs being sex-specific, or alternatively, most of the trans eQTLs could be false positives. In order to examine this further, we assembled a new set of transcripts that were called present in >1/3 of the pooled male and female expression data, thus enabling new analyses on a single set of transcripts for both sexes. We repeated the eQTL analysis six more times, once using the correct sex assignment, and five times with permuted sex assignments while preserving the number of males and females. At the LOD 4.3 threshold there were 414 and 1132 trans eQTLs in the females and males, respectively, using the correct sex assignments, with a total of 1546 trans eQTLs and 40 shared in both sexes (Table 5). If the trans eQTLs are primarily sex-specific, we would expect a large decrease in their numbers in the permuted datasets. There were on average 642 and 677 trans eQTLs in the permuted female and male datasets, respectively, with an average total of 1319 trans eQTLs and of 40 shared trans eQTLs in both sexes. Since the number of total trans eQTLs was only decreased by an average of 15% in the permuted datasets compared to the correct sex assignments, it is possible that many of the trans eQTLs are false positives. However, for the male cohort, there was an average 40% decrease in the number of trans eQTLs in the permuted datasets, suggesting that many of the male trans eQTLs may be authentic. As in the prior analysis, the majority of the cis eQTLs were shared in two new analyses (Table 5)

**Table 5:** Cis and Trans eQTLs≥LOD 4.3 in male and female mice with and without 5 gender permutations#

	***Female***	***Males***	***Shared***	***Female permuted***	***Male permuted***	***Shared permuted***
Cis eQTLs	1,328	1,442	1,005	1,154±18	1,341±9	992±37
Trans eQTLs	414	1,132	40	642±128	677±65	40±6

# based upon a pool of transcripts using the combined male and female expression data. Values for permuted data are mean+S.D. for 5 separate gender permutations.

### Sex Effects on Gene Expression

We next examined differences in gene expression levels between the male and female F_2_ macrophages. We pooled the male and female expression data, and set an arbitrary cutoff for expressed genes, in that the transcript must have been called expressed in 1/3 of the pooled samples. Altogether, there were 22,056 transcripts that met this criterion. We then performed non-parametric Mann Whitney tests to determine which of these were expressed differently in the male and female macrophages. Remarkably 6,719 transcripts (31%) were expressed differently with p-values<2.27×10^−6^, meeting the conservative Bonferroni corrected p-value of <0.05. Since the RNA under study was derived from cells cultured two weeks *ex vivo*, these sex effects on gene expression are likely attributable to X and Y chromosome dosage effects, rather than to endogenous and variable sex steroids in the F_2_ mice. About half of these (3,304) were expressed higher in female macrophages (female bias), and the other half (3,415) were expressed higher in male macrophages (male bias). These sex biased genes had a large range of fold differences between the sexes, with most having only modest effects of 1.2 to 1.5 fold, but also included 233 transcripts with 2 to 3-fold effects, 40 transcripts with 3 to 10-fold effects, and 10 transcripts with >10-fold effects (Table 6). Supplemental Table S7 gives the details for each of these sex biased transcripts ranked by fold-difference. All three female bias probes with >10-fold effects were not expressed in males and represented the same gene on the X chromosome, *Xist*. The *Xist* gene encodes a non coding but functional RNA known to play an important role in X-chromosome inactivation in females [17]; and, it has been previously identified as transcript expressed in female, but not male, mouse blastocyts [18]. Likewise, all seven male bias transcripts with >10-fold effects were not expressed in females and mapped to the Y chromosome. These seven probes represent 4 distinct genes: *Ddx3y*, encoding a DEAD box RNA helicase; *Eif2s3y*, encoding a translation initiation factor subunit; *Uty*, encoding a ubiquitously expressed tetratricopeptide repeat; and *Jarid1d*, encoding jumonji. In contrast, most of the genes regulated <10-fold effects mapped the autosomes.

**Table 6:** Number and fold-effects of transcripts with sex biased expression with p<2.27×10^−6^.

Fold effect	Female bias	Male bias	Total
1.0–1.2	1071	770	1841
1.2–1.5	1846	1579	3425
1.5–2.0	229	664	893
2–3	18	215	233
3–10	1	39	40
>10	3	7	10
Total	3304	3415	6719

Gene ontology analysis for the 3304 female biased transcripts compared to all expressed transcripts revealed many over represented classes (including regulation of metabolism, transcription, and zinc binding proteins) a few under represented classes (including the extracellular space). Supplemental Table S8 gives a full listing and P-values for the gene ontology findings of the female biased transcripts. Gene ontology analysis for the 3415 transcripts with male biased expression found over representation in the cytoplasm cellular component (p = 0.001), and the biological processes of protein transport, localization, and establishment of protein localization (all p<0.05).

3,974 (59%) of the 6,719 probes that exhibited sexually dimorphic expression were also associated with 2265 female and/or 2852 male eQTLs on the autosomes and X chromosome with LOD scores >3.0, with 74% and 78% of these classified as trans eQTLs in females and males, respectively (Supplemental Table S9). An additional 299 probes exhibited sexually dimorphic expression and had eQTLs on the Y chromosome, with 90% classified as trans eQTLs (Supplemental Table S10). Interestingly, more of these Y chromosome eQTLs exhibited female bias (166) than male bias (133), indicating a Y chromosome effect on decreasing expression of specific transcripts in males. We mapped the genomic distribution of these sex- biased eQTLs, and they basically shared the hotspot distribution that we observed for the overall female and male eQTLs distribution (Fig 1C, D).

## Discussion

In the current work, we have identified eQTLs separately from male and female bone marrow derived macrophages derived from F_2_ mice from an AKR x DBA/2 strain intercross. These studies are subject to methodological considerations for both the microarray and QTL analyses. We chose to use Microarray Suite 5.0 (Affymetrix) for array normalization and expression levels, as this method has been shown to yield results similar to those obtained using the more conservative RMA normalization procedure [19]–[21]. We also chose not to log transform the gene expression data. Although this will give relatively more weight to the data with large gene expression values, we prefer this over giving equal weight to a doubling of gene expression at the low end of the scale (e.g. 50 vs. 100 arbitrary MAS5 units), where the precision of the measure is expected to be lower and the signal approaches the background level, versus the high end of the scale (e.g. 5,000 vs. 10,000 arbitrary MAS5 units), where the precision of the measure is expected to be higher and the signal is far above the background level. Furthermore, we did not screen the Affymetrix probe sets for SNPs polymorphic between the AKR and DBA/2 strains, but based on a prior screen for polymorphic SNPs between the C57BL/6 and DBA/2 strains [19], only a small fraction of the cis eQTLs identified may be artefactual due SNPs in the probe sequence that could alter hybridization to the array. Nevertheless, some of our cis eQTLs could be due to either a polymorphic SNP overlapping the probe sequence, or a polymorphic copy number variation for the probe target sequence; and either of these would be expected to give rise to strong and highly heritable cis eQTLs. For our QTL analysis, we used the suggestive and significant LOD thresholds of 3.0 and 4.3 [16], as used in prior eQTL studies of mouse strain intercrosses [2], [12]; in addition, we performed permutation analysis to directly calculate genome wide p-values of 0.25 and 0.02, respectively, for these LOD thresholds. These genome wide p-values were the same for cis and trans eQTLs at any given LOD threshold. However, it may be argued that we are underestimating the strength of the cis eQTLs, since the genome wide permutations utilized all markers and all probesets, while only one linked marker needs to be used to test the strength of cis eQTLs. We analyzed all eQTLs in single sex cohorts, as sex has been shown be markedly affect gene expression and eQTLs in various mouse tissues [12], [13], and this strategy proved particularly important for trans eQTLs which were overwhelmingly sex-specific.

At the suggestive LOD threshold of 3.0, there were ∼3 to 4 times more trans eQTLs than cis eQTLs in both sexes. However, the average LOD score for the cis eQTLs was much higher than for trans eQTLs, as previously observed [2], presumably due to the direct effect of cis regulation being stronger than the indirect effect of trans regulation. At the significant LOD threshold of 4.3, there were instead more cis eQTLs than trans eQTLs. At this threshold, the vast majority of the cis eQTLs were retained from the suggestive threshold in both sexes, while only ∼10% of the trans eQTLs were retained from the suggestive threshold. This shift from predominantly trans eQTLs at the suggestive threshold to predominantly cis eQTLs at the significant threshold was also observed in an eQTL study of adipose tissue from a mouse strain intercross [12].

The occurrence of eQTLs hotspots resembled prior studies in which eQTL hotspots were found [1], [2]. Interestingly, the genes responsible for two trans eQTL hotspots have been identified in yeast, and both are signal transduction proteins rather than transcription factors, one is a G-protein subunit of a pheromone receptor, and the other is a protein that inactivates a transcription factor activator [22]. We found two autosomal eQTL super hotspots in the male F_2_ cohort on chromosomes 1 and 16. We identified nine candidate genes at the chromosome 1 hotspot, each with a strong cis eQTLs and whose expression is well correlated with the expression of the trans eQTLs mapped to the same locus (Table 2). Five of these nine genes have activities which suggest they could be responsible for the trans regulation of many genes. *Nif3l1* encodes a highly conserved protein that has been shown to bind to other nuclear proteins and alter their transcription factor activity [23], [24]. *Abi2* encodes an SH3 domain containing protein that binds to and modulates c-abl activity with effects on cell morphogenesis and motility [25], [26]. Little is known about *Raph1*, but it encodes a protein that contains both Ras association and plekstrin homology domains, thus it could play a role in signal transduction. *Tgfbrap1* encodes a protein that binds to TGFβ receptor 1 and plays a role in Smad-mediated signal transduction [27], [28]. *Unc50*, the homologue of the C. elegans *unc-50* gene, encodes a nuclear protein with RNA binding activity that has been shown to alter specific gene expression [29]. We identified three candidate genes at the chromosome 16 hotspot, each with a strong cis eQTL and whose expression is well correlated with the expression of the trans eQTLs mapped to the same locus (Table 3). One of these genes, *Dgcr8*, has an activity which suggests that it could be responsible for the trans regulation of many genes. *Dgcr8* encodes an RNA binding protein that associates with Drosha, and which is required for microRNA processing with potentially global effects on gene expression [30]–[32]. Further work would be required to confirm whether any of these candidates are in fact responsible for these eQTL super hotspots.

The third eQTL super hotspot in the males was on the Y chromosome. This is the first report, of which we are aware, of Y chromosome eQTLs. We were able to identify these due to the reciprocal nature of the strain intercross. At the LOD 4.3 threshold there were 334 Y chromosome eQTLs in the male cohort (almost all trans eQTLs), greatly outnumbering the 31 eQTLs on the X chromosome in the males. This indicates that the Y chromosome strain difference had a larger effect on gene expression in male bone marrow derived macrophages than the X chromosome strain difference. There were 171 female eQTLs mapped to the X chromosome at the LOD 4.3 threshold, also outnumbering the 31 male eQTLs mapped to the X chromosome, indicating that strain differences on the X chromosome were more important in regulating gene expression in female than in male macrophages.

Although the majority of the cis eQTLs at either LOD threshold were conserved between the male and female cohorts, we were surprised by the low level of sharing for the trans eQTLs, with only ∼1 to 2% of female or male trans eQTLs common to both sexes. We considered two possible interpretations of this finding: 1) the sex chromosomes play an enormous role in trans regulation of gene expression; or 2) the majority of trans eQTLs are false positives and therefore not conserved between the sexes. The evidence that supports the first interpretation is: a) the trans eQTLs were identified by the same methods that found the cis eQTLs, which are robustly conserved; and b) our genome wide p-values are based upon permutation analysis which takes into account the data structure, and thus these are likely to be good estimates. However, there is also evidence to support the second interpretation: permuting the sexes did not greatly reduce the number of total trans eQTLs, although it did reduce the trans eQTLs in males by 40%. Another group has also found that trans eQTLs are not reproducible, in this case comparing trans eQTLs discovered using an F_2_ cohort and a panel of recombinant inbred strains derived from the same two parental strains [33]. Thus, we cannot convincingly conclude at this time that the sex chromosomes are crucial in mediating trans regulation of gene expression, although our data support this concept. Our finding that 31% of the expressed genes (>6700 transcripts) had a male or female expression bias (confirmed by permutation analysis) lends support for the role of the sex chromosomes in global gene regulation.

The large degree of sex bias in gene expression that we detected in macrophages can partly be attributed to the large power we had to detect highly significant sex effects on gene expression even when the absolute effect was small (<20% difference) due to the large sample size (93 female and 114 male samples). This magnitude of sex biased gene expression was previously observed in mouse liver, adipose, muscle, and brain in a similar microarray study using a large F_2_ cohort [13]. In a study of three pools each of male and female mouse blastocyts using 6 two-dye microarrays, only 600 sex biased genes were detected [18], and we suspect this lower level of detection of sex biased gene expression is primarily due to the small sample size and lower power to detect small effects as significant. It may be argued that some of the sex bias in gene expression in the prior study of mouse liver, adipose, muscle, and brain could be environmental rather than genetic, due to the different hormonal and metabolic environment in male and female mice [12], [13]. In contrast, the current study used cells that were grown and differentiated for 2 weeks *ex vivo* prior to RNA preparation, which should increase the genetic component of the sex bias on gene expression by eliminating the differential and fluctuating hormonal environment of the donor mouse. However, we cannot exclude the possibility of long lasting effects of hormones that may alter cell development and thus gene expression profiles.

## Materials and Methods

### Mice

ApoE-deficient mice [34] on the C57BL/6 genetic background were bred ≥10 generations onto the AKR/J and DBA/2J genetic backgrounds. A strain intercross was performed using males and females from both parental strains as previously described [35]. The F_2_ generation mice were sacrificed at 16 weeks of age and bone marrow cells were isolated by lavage of the excised femurs, washed in phosphate buffered saline, plated in Dulbecco's modified Eagle's medium (DMEM) with 0.2% BSA into two 100 mm tissue culture dishes and allowed to adhere for 2 hrs at 37°C. Adherent cells were cultured in DMEM with 10% fetal bovine serum and 20% L-cell conditioned media (as a source of MCSF) for 2 weeks at which point they were confluent bone marrow derived macrophages that expressed macrophage specific transcripts [35].

### Gene Expression Profiling

Total RNA was prepared from macrophages of each mouse using RNAeasy minikits (Qiagen), converted into labeled cRNA, and hybridized to Affymetrix mouse 430v2 oligonucleotide arrays as previously described [35]. MicroArray Suite 5.0 (MAS5.0) software (Affymetrix) was used to compare the 11 perfect matched probes for each element with the mismatched probes and a call of present or absent was made using p<0.05 criteria. The luminosity of each element was normalized to the luminosity of the entire chip. Male and female F_2_ mice were analyzed separately, since sex is know to play a large role in gene expression levels in mouse tissues [12], [13]. Since we were using tissue from a strain intercross, and some transcripts might be absent or low in one of the parental strains, we limited our analysis to transcripts that were called present for at least 1/3 of the mice for each sex. The sex effect on the level of expression of each probe was compared in the combined female and male cohorts by use of the non-parametric Mann-Whitney U test, and unadjusted and Bonferroni corrected p-values were determined.

### Genome Scan and eQTL Analysis

DNA was prepared from frozen spleen of each F_2_ mouse and used for SNP genotyping on a 5K mouse SNP chip, as previously described [15], yielding 1967 SNPs on the 19 autosomes and the x chromosome. A polymorphic marker on the Y chromosome, Zfy2, was also genotyped to confirm the grandparental strain of each F_2_ mouse. Gene expression (not log transformed) and genotype data for each mouse were assembled and analyzed using the r/qtl software package [36], as previously described [15]. To calculate genome wide p-values of the LOD 3.0 and 4.3 thresholds, permutation analysis was performed within r/qtl. Y chromosome LOD scores were derived from the residual sum of squares for the null model (rss0) and the residual sum of squares for the Y chromosome effect (rss1), and calculated from the equation LOD = (n/2)×log10 (rss0/rss1), where n = the sample size.

Expressed Affymetrix probes on the 430v2 chip were batch queried to NetAffx raw data (release 21) using a custom software application (J. Bhasin, manuscript in preparation) to determine the chromosome and Mb position as well as the % identity for each BLAT alignment of the target sequence for each probe. Probes were scored uniquely mapped to the mouse genome if the % identity of the best match was ≥75% and the best match had >5% better identity than the second best match. The majority of the uniquely mapped probes had >95% identity with the best match. Probes that failed this test were assigned as ambiguous. Cis and trans eQTLs assignments were restricted to probes that were mapped uniquely. Cis eQTLs were assigned by the same custom software application if a probe's eQTL was within 20 Mb on the same chromosome as the map position of that probe. All other eQTLs for uniquely mapped probes that did not meet this criteria were assigned as trans eQTLs. eQTLs for ambiguously mapped probes were called ambiguous eQTLs. We observed very high LOD values for several trans eQTLs and did further manual curation that determined these were in fact ambiguous eQTLs due to probe mapping ambiguity. Gene ontology classifications and statistics were performed using GoStat (http://gostat.wehi.edu.au/L) [37].

### Data Access

Expression and genotype data for each mouse is available in a MIAME compliant format in the Gene Expression Omnibus (NCBI) website, accession # GSE8512.

## Supporting Information

Table S1 (2.21 MB XLS)

Table S2 (2.89 MB XLS)

Table S3 (0.02 MB XLS)

Table S4 (0.34 MB XLS)

Table S5 (0.03 MB XLS)

Table S6 (0.08 MB XLS)

Table S7 (1.72 MB XLS)

Table S8 (0.03 MB XLS)

Table S9 (1.42 MB XLS)

Table S10 (0.08 MB XLS)
